# Supplementary material for: Associations between moderate alcohol consumption, brain iron, and cognition in UK Biobank participants: Observational and mendelian randomization analyses
Source: PLoS Med. 2022 Jul 14;19(7):e1004039. doi: 10.1371/journal.pmed.1004039 (PMC9282660; doi:10.1371/journal.pmed.1004039)
Supplement: S1 Checklist — (DOCX) [file pmed.1004039.s001.docx]

**S1 checklist: STROBE checklist of recommended items to address in cohort studies**

| Item No. | Section | Checklist item | Where addressed in manuscript |
| --- | --- | --- | --- |
| 1 | **TITLE and ABSTRACT** | (a) Indicate the study’s design with a commonly used term in the title or the abstract | Title |
|  |  | (b) Provide in the abstract an informative and balanced summary of what was done and what was found | Abstract |
|  | **INTRODUCTION** |  |  |
| 2 | **Background/****rationale** | Explain the scientific background and rationale for the investigation being reported | Introduction – paragraph 4 |
| 3 | **Objectives** | State specific objectives, including any prespecified hypotheses | Introduction, paragraph 4 |
|  | **METHODS** |  |  |
| 4 | **Study design and data sources** | Present key elements of study design early in the paper | Introduction (paragraph 4), Fig 1, Methods, paragraph 1 |
| 5 | **Setting** | Describe the setting, locations, and relevant dates, including periods of recruitment, exposure, follow-up, and data collection | Methods, paragraphs 1-4 |
| 6 | **Participants** | 1. Give the eligibility criteria, and the sources and methods of selection of participants. Describe methods of follow-up | Methods, paragraph 1 |
|  |  | 1. For matched studies, give matching criteria and number of exposed and unexposed | Not applicable |
| 7 | **Variables** | Clearly define all outcomes, exposures, predictors, potential confounders, and effect modifiers. Give diagnostic criteria, if applicable | Methods, paragraphs 2-4,6, observational analyses (paragraphs 1-3) |
| 8 | **Data sources/measurement** | For each variable of interest, give sources of data and details of methods of assessment (measurement). Describe comparability of assessment methods if there is more than one group | Methods, paragraphs 2-4,6 |
| 9 | **Bias** | Describe any efforts to address potential sources of bias | Methods, observational analyses (confounding bias) |
| 10 | **Study size** | Explain how the study size was arrived at | SFig 1 |
| 11 | **Quantitative variables** | Explain how quantitative variables were handled in the analyses. If applicable, describe which groupings were chosen and why | Methods, paragraph 3-7 |
| 12 | **Statistical methods** | (a) Describe all statistical methods, including those used to control for confounding | Methods, observational analyses (paragraph 1) |
|  |  | (b) Describe any methods used to examine subgroups and interactions | Methods, observational analyses (paragraph 3,4) |
|  |  | (c) Explain how missing data were addressed | Methods, paragraph 1 |
|  |  | (d) If applicable, explain how loss to follow-up was addressed | Not applicable |
|  |  | (e) Describe any sensitivity analyses | Methods, observational analyses (paragraph 2,3) |
|  | **RESULTS** |  |  |
| 13 | **Participants** | 1. Report numbers of individuals at each stage of study—eg numbers potentially eligible, examined for eligibility, confirmed eligible, included in the study, completing follow-up, and analysed | SFig 1 |
|  |  | (b) Give reasons for non-participation at each stage | SFig 1 |
|  |  | (c) Consider use of a flow diagram | SFig 1 |
| 14 | **Descriptive data** | (a) Give characteristics of study participants (eg demographic, clinical, social) and information on exposures and potential confounders | Results, paragraph 1, Table 1, STable 2 |
|  |  | (b) Indicate number of participants with missing data for each variable of interest | SFig 1 |
|  |  | (c) Summarise follow-up time (eg, average and total amount) | Relevant for cognition online only (in abstract and methods, clinical measures) |
| 15 | **Outcome data** | Report numbers of outcome events or summary measures over time | Results, Table 1 |
|  |  | c) If relevant, consider translating estimates of relative risk into absolute risk for a meaningful time period | Not applicable |
|  |  | d) Consider plots to visualize results (e.g. forest plot, scatterplot of associations between genetic variants and outcome versus between genetic variants and exposure) | Fig 4 & 6 |
| 15 | **Main results** | (*a*) Give unadjusted estimates and, if applicable, confounder-adjusted estimates and their precision (eg, 95% confidence interval). Make clear which confounders were adjusted for and why they were included | Results, paragraph 4 |
|  |  | 1. Report category boundaries when continuous variables were categorized | Results, Table 1-2 |
|  |  | (*c*) If relevant, consider translating estimates of relative risk into absolute risk for a meaningful time period | Results, paragraph 4 |
|  |  | b) Report results from other sensitivity analyses or additional analyses | SFig 2, SFig 3, SFig 7, SFig 8 |
| 17 | **Other analyses** | Report other analyses done—eg analyses of subgroups and interactions, and sensitivity analyses | Results, paragraphs 2-4, SFig 3-10 |
|  | **DISCUSSION** |  |  |
| 18 | **Key results** | Summarise key results with reference to study objectives | Discussion, paragraph 1 |
| 19 | **Limitations** | Discuss limitations of the study, taking into account sources of potential bias or imprecision. Discuss both direction and magnitude of any potential bias | Discussion, paragraphs 7,8 |
| 20 | **Interpretation** | Give a cautious overall interpretation of results considering objectives, limitations, multiplicity of analyses, results from similar studies, and other relevant evidence | Discussion throughout and specifically conclusion |
| 21 | **Generalisability** | Discuss the generalisability (external validity) of the study results | Discussion, paragraphs 5 & 8 |
|  | **OTHER INFORMATION** |  |  |
| 22 | **Funding** | Give the source of funding and the role of the funders for the present study and, if applicable, for the original study on which the present article is based | Financial disclosure |
