# Supplementary figures and images for: Associations between moderate alcohol consumption, brain iron, and cognition in UK Biobank participants: Observational and mendelian randomization analyses
Source: PLoS Med. 2022 Jul 14;19(7):e1004039. doi: 10.1371/journal.pmed.1004039 (PMC9282660; doi:10.1371/journal.pmed.1004039)

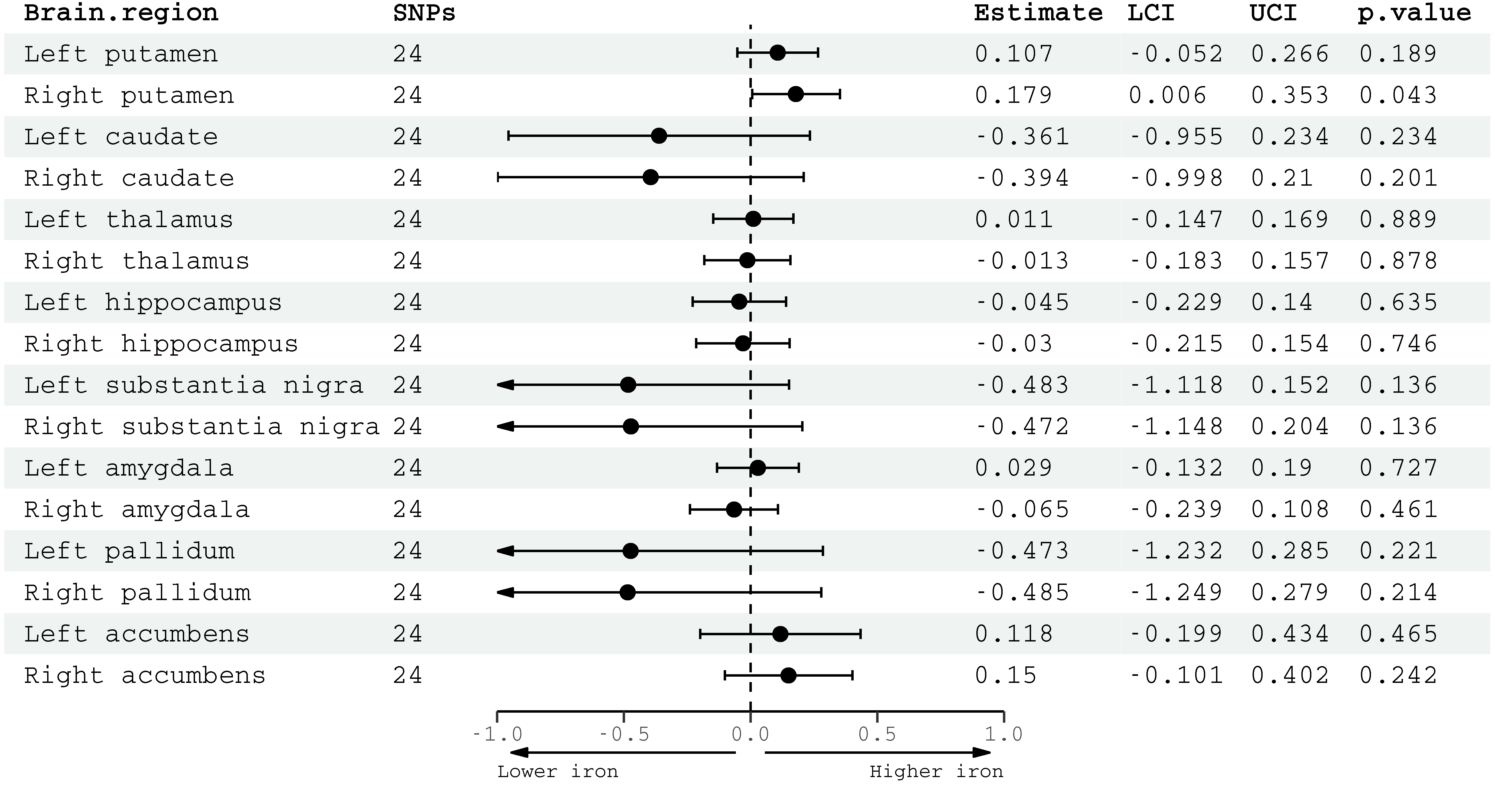

Supplement: S3 Fig — MR estimates (2-sample design) for associations between genetically predicted alcohol use disorder (Million Veterans Program and Psychiatric Genomics Consortium) and susceptibility imaging-derived phenotypes (UK Biobank) in inverse-variance weighted analysis. LCI, lower confidence interval; MR, mendelian randomization; SNP, single nucleotide polymorphism; UCI, upper confidence interval. (PNG) [file pmed.1004039.s005.png]

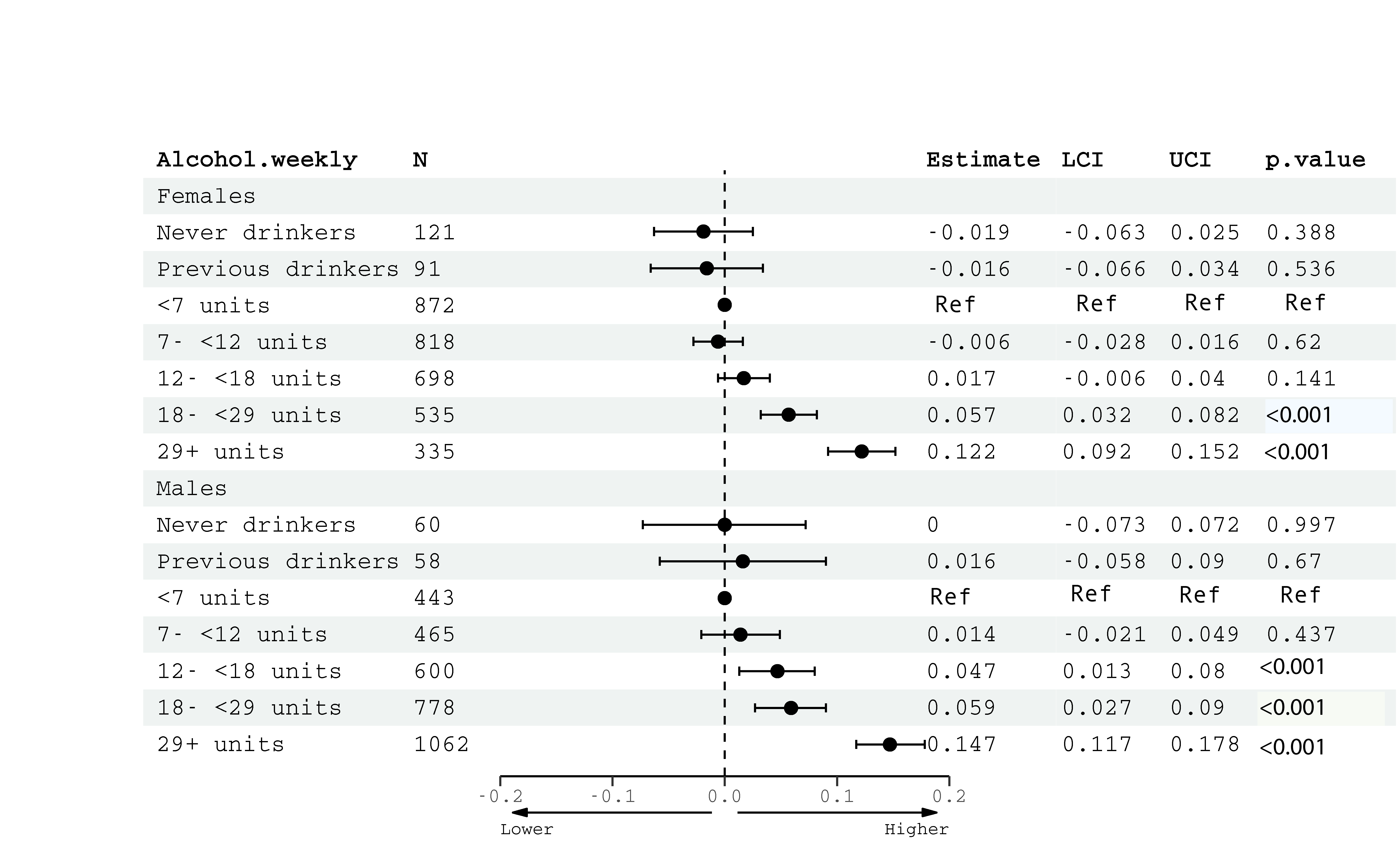

Supplement: S4 Fig — Reference group is those drinking <7 units (56 g) weekly. Estimates generated from regression models adjusted for: age, educational qualifications, Townsend Deprivation Index, household income, historical job code, smoking, imaging site, diabetes mellitus, BMI, blood pressure, cholesterol, dietary iron, rs1800562, rs1799945, and rs855791. BMI, body mass index; LCI, lower confidence interval; UCI, upper confidence interval. (PNG) [file pmed.1004039.s006.png]

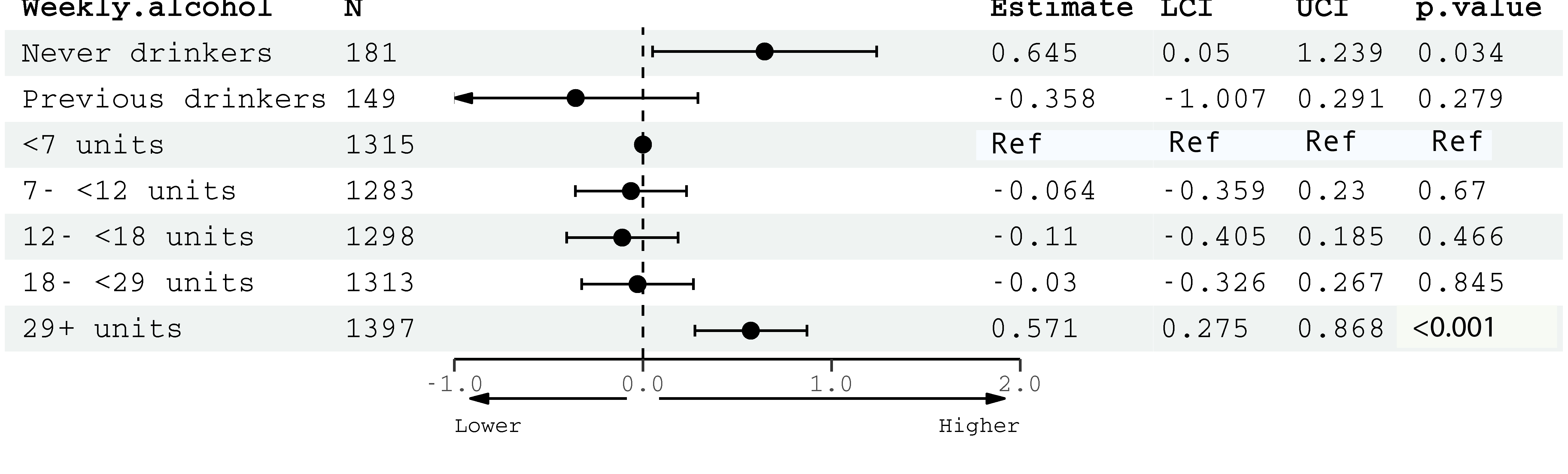

Supplement: S5 Fig — Estimates are adjusted for age, sex, smoking, BMI, cholesterol, blood pressure, diabetes, educational qualifications, Townsend Deprivation Index, household income, and historical job type. BMI, body mass index; LCI, lower confidence interval; UCI, upper confidence interval. (PNG) [file pmed.1004039.s007.png]

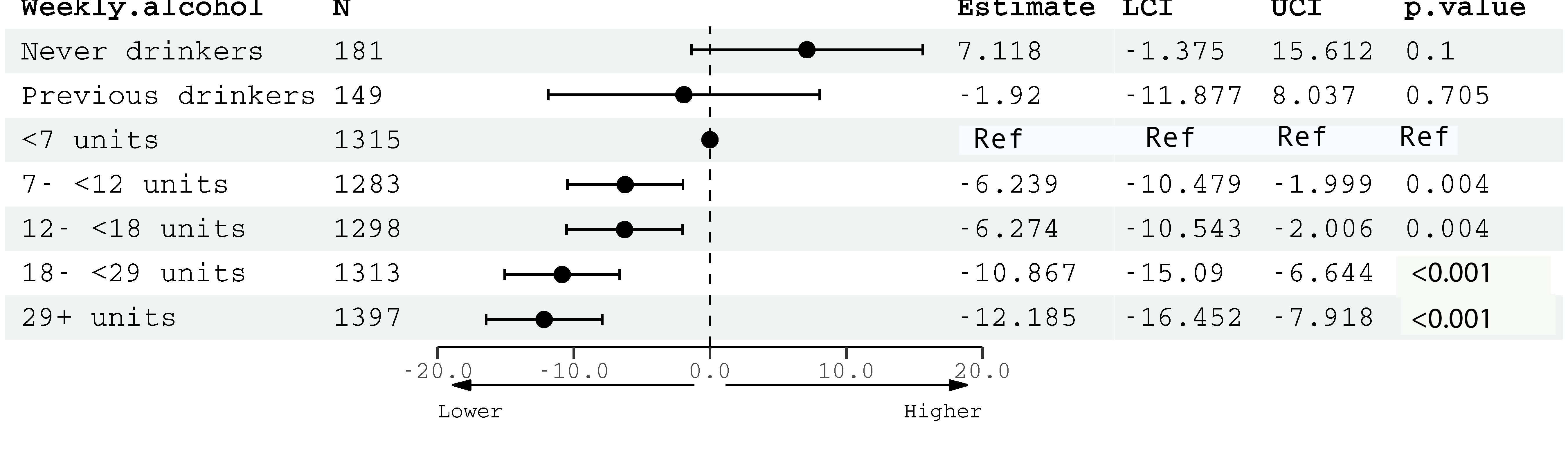

Supplement: S6 Fig — Estimates are adjusted for: age, sex, smoking, BMI, cholesterol, blood pressure, diabetes, educational qualifications, Townsend Deprivation Index, household income, and historical job type. All estimates are within the normal reference range [88]. BMI, body mass index; LCI, lower confidence interval; UCI, upper confidence interval. (PNG) [file pmed.1004039.s008.png]

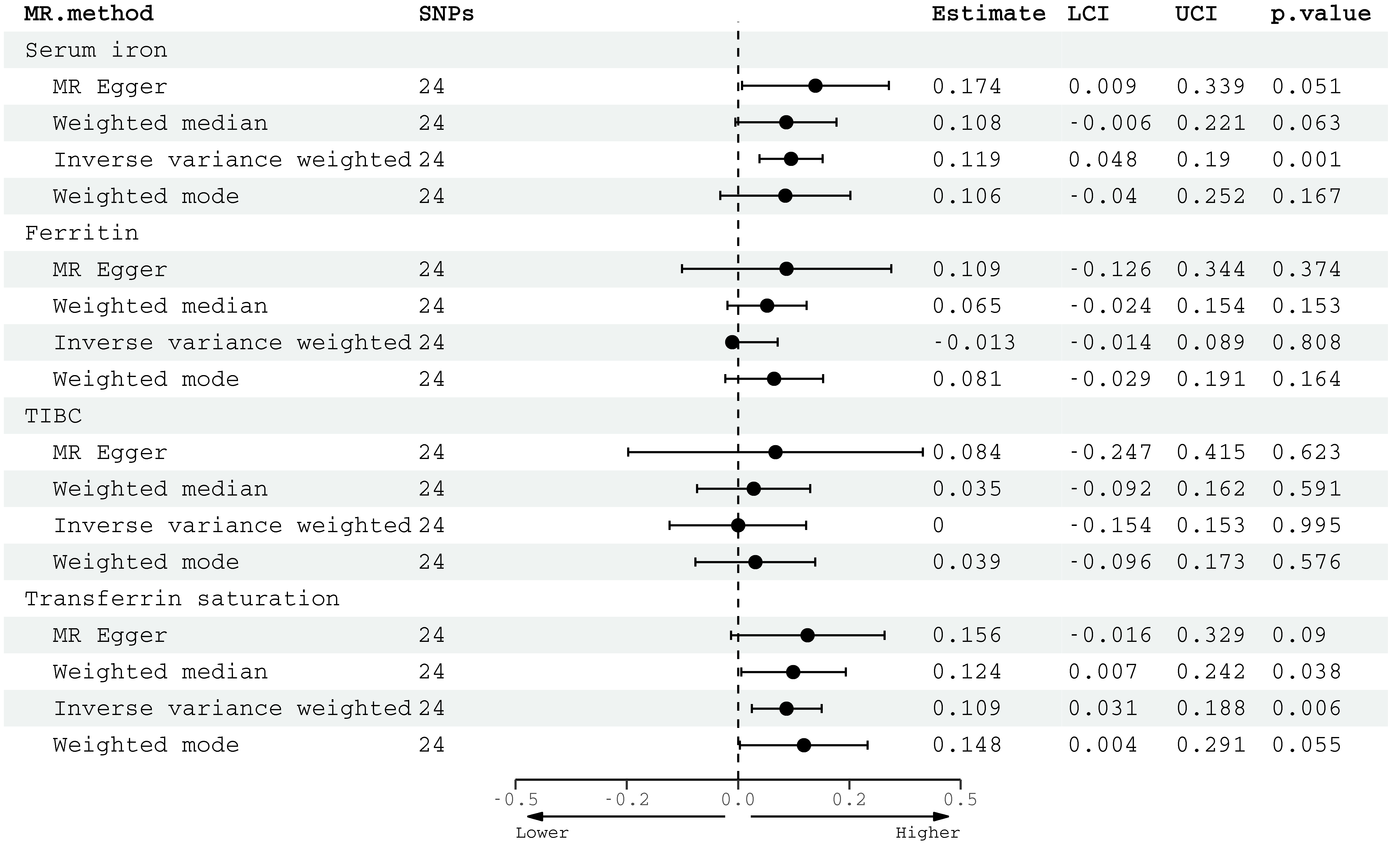

Supplement: S7 Fig — Genetic associations of alcohol use disorder generated in the Million Veterans Program and Psychiatric Genomics Consortium and of serum markers of iron homeostasis from deCODE, INTERVAL, and the Danish Blood Donor Study. LCI, lower confidence interval; MR, mendelian randomization; SNP, single nucleotide polymorphism; TIBC, total iron binding capacity; UCI, upper confidence interval. (PNG) [file pmed.1004039.s009.png]

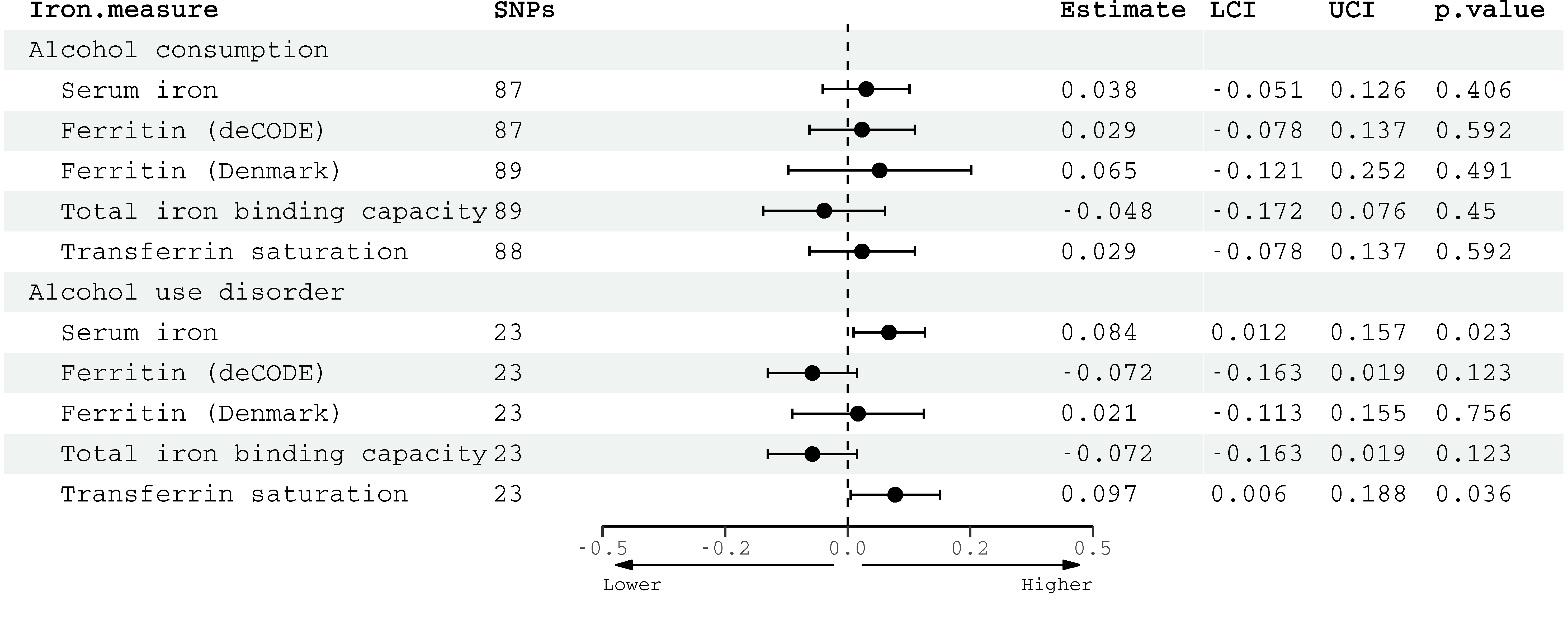

Supplement: S8 Fig — Genetically predicted alcohol consumption was log-transformed drinks per week, generated from GWAS and Sequencing Consortium of Alcohol and Nicotine. Genetic associations with alcohol use disorder were generated from the Million Veterans Program and Psychiatric Genomics Consortium. Genetic associations with serum iron markers were calculated in cohorts that did not adjust for alcohol in their genome-wide association study (DECODE unless otherwise marked). LCI, lower confidence interval; SNP, single nucleotide polymorphism; UCI, upper confidence interval. (PNG) [file pmed.1004039.s010.png]
