# Supplementary material for: Associations between moderate alcohol consumption, brain iron, and cognition in UK Biobank participants: Observational and mendelian randomization analyses
Source: PLoS Med. 2022 Jul 14;19(7):e1004039. doi: 10.1371/journal.pmed.1004039 (PMC9282660; doi:10.1371/journal.pmed.1004039)
Supplement: Attachment — Submitted filename: Plos Medicine rebuttal 7.4.22.docx [file pmed.1004039.s022.docx]

**Plos Medicine rebuttal**

**Requests from the editors:**

1. Competing Interests: Please add this statement to the manuscript's Competing Interests: SBu is a paid statistical consultant on PLOS Medicine's statistical board.

We have added this statement.

2. Data Availability Statement: The Data Availability Statement (DAS) requires revision. For each data source used in your study:

a) If the data are freely or publicly available, note this and state the location of the data: within the paper, in Supporting Information files, or in a public repository (include the DOI or accession number).

b) If the data are owned by a third party but freely available upon request, please note this and state the owner of the data set and contact information for data requests (web or email address). Note that a study author cannot be the contact person for the data.

c) If the data are not freely available, please describe briefly the ethical, legal, or contractual restriction that prevents you from sharing it. Please also include an appropriate contact (web or email address) for inquiries (again, this cannot be a study author).

Please also indicate how any scripts necessary for analysis may be accessed.

*“Imaging and observational data underlying the results presented are available from the UK Biobank upon successful application (*[*https://www.ukbiobank.ac.uk/enable-your-research/apply-for-access*](https://www.ukbiobank.ac.uk/enable-your-research/apply-for-access)*). Genetic summary statistics for serum iron measures are freely available (*[*https://www.decode.com/summarydata/*](https://www.decode.com/summarydata/)*), as are GSCAN summary statistics (*[*https://genome.psych.umn.edu/index.php/GSCAN*](https://genome.psych.umn.edu/index.php/GSCAN)*). Summary statistics for alcohol use disorder are available upon application through dfGaP at accession no. phs0016732.v3.p1 (*[*https://www.ncbi.nlm.nih.gov/projects/gap/cgi-bin/study.cgi?study_id=phs001672.v3.p1*](https://www.ncbi.nlm.nih.gov/projects/gap/cgi-bin/study.cgi?study_id=phs001672.v3.p1)*). Requests for code can be made to the authors.”*


3. Throughout: Please include line numbers running throughout the text.

Line numbers now included.

4. Abstract: Please structure your abstract using the PLOS Medicine headings (Background, Methods and Findings, Conclusions).

The abstract has been structured according to PLOS Medicine headings.

5. Abstract: Methods and Findings: Please mention the years during which the study took place, when imaging took place relative to baseline assessments, length of follow up for the cognition assessment, and details of main outcome measures.

We have added details to the abstract of the timing of imaging, cognitive follow up and explicitly state the main outcome measures as follows:

*“The main outcomes were susceptibility (χ) and T2*, indices of iron”.*

*“Imaging was performed 9.6±1.1 years after study baseline (2006-10).”*

*“Cognition was assessed at the time of scan and in online follow-up (mean 5.82±0.86 years after baseline).”*

We also now state effects on specific outcome measures in the Findings section:

*“Alcohol consumption was associated with markers of higher iron (higher χ and lower T2*).”*

6. Abstract: Methods and Findings: Please quantify the main results presented with both 95% CIs and p values.

P values (in addition to 95% CI) have been added to all results in the abstract.

7. Abstract: Methods and Findings: In the last sentence of the Abstract Methods and Findings section, please describe the main limitation(s) of the study's methodology.

We have added the following sentence to the Methods and Findings:

*“The main limitations of the study include χ and T2* reflecting changes in myelin as well as iron, alcohol was self-reported, and MR estimates can be influenced by genetic pleiotropy.”*

8. Abstract: Conclusions: Here, and throughout, please temper assertions such as “This study represents the largest…” with “To the best of our knowledge…” or similar. Please interpret the study based on the results presented in the abstract, emphasizing what is new without overstating your conclusions.

We have amended the conclusions and interpretation of our results as follows:

*“To the best of our knowledge, this study represents the largest investigation of moderate alcohol consumption and iron homeostasis to date. Levels of alcohol consumption above 7 units weekly were associated with higher iron levels in the brain. Iron accumulation represents a potential mechanism for alcohol-related cognitive decline.”*

9. Author summary: At this stage, we ask that you include a short, non-technical Author Summary of your research to make findings accessible to a wide audience that includes both scientists and non-scientists. The Author Summary should immediately follow the Abstract in your revised manuscript. This text is subject to editorial change and should be distinct from the scientific abstract. Please see our author guidelines for more information: [https://journals.plos.org/plosmedicine/s/revising-your-manuscript#loc-author-summary](https://journals.plos.org/plosmedicine/s/revising-your-manuscript#loc-author-summary)

**Why Was This Study Done?**

- There is growing evidence that even moderate alcohol consumption negatively impacts the brain, but the mechanisms underlying this are unclear.
- One possibility is that accumulation of iron in the brain could contribute, as higher brain iron has been described in numerous neurodegenerative conditions including Alzheimer’s and Parkinson’s disease.
- To the best of our knowledge, there have been no studies investigating if brain iron levels differ by level of alcohol consumption.

**What Did the Researchers Do and Find?**

- In 20,965 participants in a UK cohort study, we explored relationships between self-reported alcohol consumption and brain iron levels, measured using magnetic resonance imaging.
- We assessed the association of alcohol intake with blood and liver iron, and the clinical consequences of higher brain iron.
- Alcohol consumption above 7 units (56g) weekly was associated with markers of higher iron in the basal ganglia, which in turn associated with worse cognitive function.
- These observational findings were further supported by analyses using genetic variants as proxies for alcohol consumption.

**What Do These Findings Mean?**

- Moderate alcohol consumption can lead to higher iron levels in the brain.
- Brain iron accumulation represents a potential mechanism for alcohol-related cognitive decline.
- Key limitations are that changes in myelin may also alter imaging markers, alcohol intake was self-reported, and limited generalizability.

  10. Introduction: Please avoid vague statements such as “...there are huge public health implications.”

The statement has been amended to:

*“Given the high prevalence of moderate drinking, even small causal associations have substantial population impact [3].”*

11. Introduction: Please conclude the final paragraph of the Introduction with a clear description of the study’s objectives (in paragraph form, rather than as a numbered list).

The list has been revised to paragraph form as follows:

*“Our objective was to characterise the dose-response relationship of alcohol consumption and brain iron, in observational and Mendelian randomization frameworks. Furthermore, we sought to investigate whether alcohol influences brain iron via changes in systemic iron levels. Lastly, we explored whether higher brain iron represents a potential pathway to alcohol-related cognitive deficits.”*

12. Methods: Participants: Please clarify if participants with dementia at baseline were excluded. Please provide more detail on how those participants invited for brain/abdominal imaging were selected.

No participants with complete imaging and other data had dementia. We now clarify this:

*“No exclusions were made on the basis of dementia diagnosis as no participants with complete data had dementia at the time of imaging.”*

13. Methods: Alcohol consumption: Please clarify that only the current drinkers were categorized into quintiles (excluding former/never drinkers).

This has been clarified as follows:

*“To directly compare associations with brain iron at different levels of alcohol intake weekly consumption was categorized into quintiles in current drinkers…”*

14. Methods: Brain imaging: “The fourteen regions correspond to left and right of seven subcortical structures.” Please list the regions, with rationale for why they were selected. Please clarify if data from left/right substantia nigra were available only for QSM (as it seems there were 16 total QSM IDPs).

We now clarify which regions were included (specifying the substantia nigra was only available for QSM), and explain these are the available ROIs from the structural image:

*“The 14 regions correspond to left and right of the seven subcortical structure ROIs obtained from the T1 image: putamen, caudate, hippocampus, amygdala, pallidum, thalamus, accumbens. Two additional IDPs were calculated from QSM (left and right substantia nigra) which were not available for T2*.”*

15. Methods: Genetic variants: For each data source, please describe the study design, size, and the underlying population. Please provide relevant details on selection of the genetic variants. Please consider including a supporting information table to describe sources of data.

We now include a **STable 1** detailing all of the cohorts analyzed in the GWAS from which summary statistics were used in MR analyses. We include the following in the methods about selection of genetic variants:

*“Genetic instruments for alcohol, susceptibility and serum iron markers were selected based on the sentinel variants at genome-wide significant loci (p<5x10^-8^) reported in the largest publicly available European ancestry genome-wide association studies.”*

16. Methods: Clinical measures: Please clarify in the text which elements of the cognitive battery were assessed at baseline and at follow up. Please clarify the time point of the follow up assessment. Please describe and reference how prospective memory and fluid intelligence were assessed. Please also clarify how the subset of 100,000 participants were invited for the online follow up trail making test assessment.

We now clarify in the text elements of baseline, imaging and follow up cognitive testing, including timing, how they were assessed and how individuals were invited for the follow up as follows:

*We utilized the expanded cognitive battery performed on the subset of participants who underwent imaging, which included: trail-making tests (TMT A & B), prospective memory (incorrect or correct on 1^st^/2^nd^ attempt on a shape task), and fluid intelligence (sum of correct answers on questions designed to assess logic and reasoning) [33]. At baseline only fluid intelligence, prospective memory, digit span and pairs matching were administered. A larger subset of the wider UKB study (~100k) was invited by email to undertake further web-based questionnaires at online follow up (mean 5.82±0.86 years after study baseline). These included: TMT, fluid intelligence, digit span, pairs matching and symbol digit substitution.”*

17. Statistical analyses: Did your study have a prospective protocol or analysis plan? Please state this (either way) early in the Methods section.

a) If a prospective analysis plan (from your funding proposal, IRB or other ethics committee submission, study protocol, or other planning document written before analyzing the data) was used in designing the study, please include the relevant prospectively written document with your revised manuscript as a Supporting Information file to be published alongside your study, and cite it in the Methods section. A legend for this file should be included at the end of your manuscript.

b) If no such document exists, please make sure that the Methods section transparently describes when analyses were planned, and when/why any data-driven changes to analyses took place.

c) In either case, changes in the analysis-- including those made in response to peer review comments-- should be identified as such in the Methods section of the paper, with rationale.

We have added this to the Methods section:

*“As our study was conducted using existing resources to test an a priori hypothesis, we did not publish a pre-specified analysis plan before conducting analyses between November 2021 and January 2022.”*

18. Statistical analyses: Observational: Please clarify how each covariate was assessed, categorized, and incorporated into analyses.

We now give details of covariates. As there were >500 image-related confounds, we reference the detailed paper for these:

*“Covariates previously associated with alcohol intake or iron levels and image-related confounders were included in the models. Age in years, sex, smoking status (reported as categorical variable: never/previous/current), body mass index (calculated from measured height and weight), blood pressure (automated measurement), cholesterol (from blood sample) were assessed at baseline. Educational qualifications were self-reported in categories, as was household income. Historical job type was coded according to the Standard Occupational Classification 2000 [35], and Townsend Deprivation Index used as a continuous measure of deprivation based on census information. Diabetes was coded as a binary variable from linked electronic health records (any code for non-insulin dependent, insulin dependent, or nonspecified diabetes). The full set of imaging confounds was used as recently proposed [34] which include imaging site (3 sites), head motion, head position.”*

19. Statistical analyses: Observational: Please clarify the numbers of participants with diet/supplement data available, and whether this was missing from other participants, or if this was evaluated in only an invited subset (and how this subset was selected): “For the subset with available data, dietary factors (meat, fish, bread, fruit and vegetable consumption) and dietary iron supplementation were also included as covariates, to test whether differences in diets according to alcohol intake were driving associations.” Please also clarify how diet and supplement data were assessed in participants, and ensure that the results includes the number of participants for which these measures were available in a Table summarizing the characteristics of this sub-sample.

We now clarify that the smaller subset with dietary data was due to missing data:

*“For the subset* ***with no missing data****, dietary factors…”*

We detail how diet data was ascertained:

*“Diet and supplement data were assessed* ***at baseline via the questionnaire****.”*

Numbers of participants with diet and iron supplementation data can now be ascertained in the flowchart (**SFigure 1**) and characteristics of this sub-sample is now given in **STable 7**.

20. Statistical analyses: Observational: Please clarify if participants were excluded based on having an iron chelation prescription: “No included subjects reported iron chelator prescription.”

Now clarified:

*“No subjects were excluded on the basis of iron chelator prescription as none were documented.”*

21. Statistical analyses: Observational: Please clarify how the repeated assessment of TMT (at baseline and follow up) was incorporated into the analysis.

TMT was only performed at online follow-up and scan, not baseline. We clarify in the methods that separate models were used to test associations:

*“Separate models were used to test cognitive performance at each time point.”*

22. Statistical analyses: Causal mediation analysis: Please provide additional detail of the CMA. Please specify, the details of the regression model, the inclusion of variables, confounders, and interaction terms, assumptions, and how missing data were dealt with.

We have added further details to the causal mediation analysis methods as follows:

*“CMA was run on participants with complete data. Two separate multiple linear regression models were run. First, with putamen susceptibility as the dependent variable, with alcohol, age, sex, smoking, blood pressure, iron genetic risk score, BMI, cholesterol and liver iron as covariates. In the second model, the mediator, liver T2*, was the dependent variable, with all covariates as in the first model. CMA was then run using nonparametric bootstrapping to generate confidence intervals (1000 simulations). Assumptions in CMA include no unmeasured confounding between the exposure and outcome, between the exposure and mediator, or between the mediator and the outcome.”*

23. Statistical analyses: Mendelian randomization: Please provide complete details for the Mendelian randomization methods used and report the study according to STROBE-MR guidelines. Please mention the three core IV assumptions for the main analysis (relevance, independence and exclusion restriction). Please mention how missing data were dealt with.

We now report methods and studies according to the STROBE-MR guidelines (**S2 Checklist**). Specifically, MR assumptions have been added:

*“There are three key assumptions: 1) genetic variants are robustly associated with the exposure, 2) they share no common cause with the outcome, and 3) that genetic variants only affect the outcome through the exposure.”*

24. Statistical analyses: Mendelian randomization: Please comment on risk of bias attributable to participant overlap between the datasets used.

We have amended the methods to clarify no overlapping samples were used:

*“Genetic associations were used from the sample excluding UK Biobank, to avoid bias towards the observational estimate for overlapping samples.”*

25. Reporting: Please ensure that the observational component of the study is reported according to the STROBE guideline, and include the completed STROBE checklist as Supporting Information. Please ensure that the Mendelian randomization portion of the study is reported according to the STROBE-MR guideline, and include the completed STROBE-MR checklist as Supporting Information.

Both STROBE (**S1 Checklist**) and STROBE-MR (**S2 Checklist**) checklists are now included as supporting information.

Please add the following statement, or similar, to the Methods: "This study is reported as per the Strengthening the Reporting of Observational Studies in Epidemiology (STROBE) guideline, including guidelines specific for Mendelian randomization studies (S1 and S2 Checklist)." or similar.

The STROBE-MR guideline can be found here: [https://www.strobe-mr.org/](https://www.strobe-mr.org/)

When completing the checklists, please use section and paragraph numbers, rather than page numbers.

Done.

26. Results: Please report the complete results from the study, including the results for each brain region. Currently, results are presented for individual brain regions selectively. PLOS does not permit “data not shown” and request that results for each brain region be provided (at least in the Supporting Information).

Results for all brain regions are now provided in supplementary tables.

27. Results: For all observational analyses, please present the results of both unadjusted and adjusted analyses. Please consistently present both 95% CIs and p values in the text and tables.

We now include a supplementary table detailing the adjusted and unadjusted analyses results for QSM and T2*. 95% CI and p values are cited in the text and tables.

28. Results: Associations with brain iron: Please remove the duplicate text here “0.09 standard deviation (S.D.) [95% confidence interval 0.07 to 0.10]), caudate (β=0.06 [0.04 to 0.07]) and substantia nigra (β=0.04 [0.02 to 0.05]), and lower iron in the thalami (β= -0.05 [-0.06 to -0.03” and please report p values for these associations.

Duplicate text has been removed and p values reported.

29. Results: It is not clear why the results from left putamen are being highlighted. Please provide similar results (e.g. Figure 2A) for all brain regions.

We now combine results for all brain regions into one plot rather than highlighting individual regions (**Figure 3**).

30. Results: “A sensitivity analysis with finer categorisation of alcohol intake confirmed that associations were not observed at lower intakes (SFigure 1).” Please clarify if this means intakes lower than 7 units.

Now clarified:

*“A sensitivity analysis with finer categorisation of alcohol intake revealed that associations were not observed at lower intakes than 7 units (****STable 4****).”*

31. Results: “Menopause status did not associate with putamen susceptibility in females, nor did it not alter associations between alcohol and susceptibility (STable 1).” Please clarify if associations by menopause status was investigated for all brain regions.

As per the earlier request, we now include (as a supplementary table) associations between menopause status and susceptibility of all brain regions, and associations between alcohol and susceptibility controlling for menopause status. We clarify this in the text:

“*Menopause status did not associate with susceptibility in most brain regions in females, with the except of slightly higher left thalamus (beta=0.16, 95% CI: 0.04 to 0.27, p=0.01) and left hippocampus (beta= 0.17, 95% CI: 0.05 to 0.29, p=0.006) in females who had hysterectomies compared to pre-menopausal females (****STable 5****). Controlling for menopause status did not alter associations between alcohol and susceptibility for any brain region.”*

32. Results: “In the subset with complete data, neither adjustment for dietary factors, including frequency of red meat consumption (SFigure 8), nor dietary iron supplementation (SFigure 9) changed the pattern of associations.” Please provide a comparison of characteristics for those with compared to without these data available (including information on diet and supplementation).

We now include **STable 6** with baseline characteristics of participants with data on diet and iron supplementation data, which can be compared to **Table 1** which summarizes characteristics for the wider UKB and imaging sub-samples.

33. Results: Pathways from alcohol to brain iron: Please clarify if the mediation analysis was carried out for each brain region. It is not clear if the example results presented are from the left or right putamen. Please provide the results of the mediation analysis for each brain region in the study in the supporting information files.

We have amended **Figure 5** to clarify the example results shown are for the left putamen. Mediation analysis for each brain region in the study is now included in **STable 8**.

34. Results: “Alcohol consumption (>11 units) was associated with higher liver iron measured by MRI, a robust marker of systemic iron stores” Please provide the results supporting this finding, with 95% CIs and p values.

The text (and **SFigure 12**, now **SFigure 4**) have been revised accordingly:

*“Alcohol consumption greater than 11 units weekly was associated with higher liver iron measured by MRI, a robust marker of systemic iron stores in males (*β *0.05, 95% CI: 0.01 to 0.08, p=0.006). In females, higher liver iron was observed at intakes greater than 17 units weekly (*β *0.06, 95% CI: 0.03 to 0.08, p=7.89x10^-6^) (****SFig 4****).”*

35. Results: “Higher putamen, caudate and hippocampal susceptibility were associated with greater age-related slowing of executive function, and higher putamen and caudate susceptibility with greater age-related differences in fluid intelligence (Figure 6 (A-C) & STable 3).” Age-related slowing seems to imply you are referring to the repeated assessment of the Trail Making Test, while the figures seem to describe cross-sectional associations. Please revise to clarify. Please also clarify why results for putamen, caudate, and hippocampus are mentioned- and whether findings differed bilaterally (i.e. the choice to include left vs. right in Figure 6).

We have revised this section to clarify we are referring to age x susceptibility interactions, for example:

*“Additionally, there were* ***significant interactions between age and putamen susceptibility***  *(TMT A: duration β=0.006, 95% CI: 0.003 to 0.008, p=3.31x10^-7^; TMT B duration β=0.004, 95% CI: 0.002 to 0.006, p=6.58x10^-^4)…”*

We also specify whether associations were bilateral or which side was associated, for example:

*“Additionally, there were significant associations between age and* ***bilatera****l putamen ….* *and* ***right*** *hippocampal (TMT A: β= 2.95 x10^-3^, 95% CI: 8.43x10^-4^ to 5.05x10^-3^,p=6.07x10^-3^) susceptibility.”*

36. Results: Clinical relevance of elevated brain iron: Please report p values for all results reported, alongside the 95% CIs. Please explain when findings for left vs right hemisphere brain regions are described, whether findings are consistent bilaterally.

P values are now reported for all results. We now explicitly state whether associations were with left vs. right sides as described above.

37. Discussion: Please present and organize the Discussion as follows: a short, clear summary of the article's findings; what the study adds to existing research and where and why the results may differ from previous research; strengths and limitations of the study; implications and next steps for research, clinical practice, and/or public policy; one-paragraph conclusion.

The discussion has been revised according to the requested format.

38. Discussion: Key findings: Please differentiate between observational evidence, and evidence derived from genetically predicted alcohol use. Please describe as “evidence supporting a causal relationship between AUD and higher serum iron” or similar.

This paragraph has been revised to clarify which associations were observational and which genetic:

*“Alcohol consumption, including at low levels, was* ***observationally associated*** *with higher brain iron in multiple basal ganglia regions. There was some evidence supporting a* ***causal relationship between genetically predicted*** *alcohol consumption and putamen and hippocampus susceptibility, although this did not survive multiple testing correction. Alcohol was associated with both higher liver iron, an index of systemic iron load, and* ***genetically predicted*** *AUD associated with genetically predicted serum iron markers.”*

39. References: Please use the "Vancouver" style for reference formatting, and see our website for other reference guidelines [https://journals.plos.org/plosmedicine/s/submission-guidelines#loc-references:](https://journals.plos.org/plosmedicine/s/submission-guidelines#loc-references:)

References are now reported in Vancouver style.

40. Table 1: Please define units of alcohol in the legend. Please also describe “higher degree” in the legend. In the Methods, please describe how education level was categorized. Please report on all relevant variables incorporated into the analyses.

We now define alcohol units in the legend as:

*“Alcohol is measured in UK units weekly. 1 unit = 8 grams ethanol.”*

**Table 1** now includes key baseline variables. We have amended ‘higher degree’ to clarify university or college degree (self-reported).

41. Figure 2: Please include a larger version of Panel A, rather than including this as an inset. Please include p values for Panel A. For the graph, it would be helpful to include more descriptive terms with the axis labels. Please also explain the color code system for the markers.

What was previously Panel A is now included as a separate **Figure 3**, with p values.

We now explain in the **Figure 2** legend that the dots corresponding to FDR/Bonferroni-significant estimates are coloured, with colours matching brain region labels for ease of assessing which regions have significant results. We have amended the axis labels for clarity.

42. Figure 3: Please also report p values.

P values are now also reported.

43. Figure 5: Please also report p values.

P values are now also reported.

44. Supporting Information: Please make sure that each Supporting Information Figure/Table has a title and a legend.

All main manuscript and supplementary Figures and Tables now have titles and legends.

45. Figures S1 - S9: Please provide p values. Please provide the results of sensitivity analyses for all brain regions included in the study (by octiles of alcohol consumption, with adjustments for diet and iron supplementation).

S1-9 have been combined (into **Figure 3**) as requested in response to point 46 below, with p values displayed. The sample with complete data on diet and iron supplementation is much smaller (n=6516), so we have presented brain regions without this adjustment in the main manuscript, but now include **STable 7** controlling for these factors in all brain regions. We also include **STable 4** for all brain regions by octiles of alcohol consumption.

46. Figure S10: Please move the inset (A) to below the graph, and increase the size. Please include p values with the 95% CIs reported in the panel. The rationale for illustrating findings from right putamen are not clear. Please provide the result for the T2* derived analyses for each brain region in the study, in addition to the right putamen, especially given that the left putamen was selected for illustration in Figure 2.

Results for all brain regions are now shown for the T2* analyses in **STable 3**, rather than highlighting the right putamen. Given the large amount of supplementary tables now, we have decided to retain only these T2* analyses rather than alcohol quintiles in addition.

47. Figure S11: Please include p values and please include the results for each brain region included in the study.

Figure S11 (now **SFigure 2**) has been revised to include robust MR estimates for all brain regions (with p values).

48. Figure S12, S13, S14, S15, S16: Please include p values.

P values are now included in all revised Figures.

49. Table S1: Please report exact p values, please do not report p<0.05. Please include the data for each brain region in the study.

We now provide **STable 5** giving results for all brain regions with control for menopause status, with exact p values.

50. Table S2: It may be helpful to present these data similar to Figure 3 in the main text. Please report exact p values rather than p<0.05.

Data from **STable 2** is now presented as a forest plot (**SFigure 3**), including exact p values.

51. Table S3 and Table S4: Please report exact p values rather than p<0.05, etc. Please present associations for all brain regions included in the study.

Results from all brain regions are now shown, with exact p values in **STable 9**.

Comments from the reviewers:

**Reviewer #1:** This is an interesting paper testing the somewhat novel hypothesis that alcohol causes cognitive decline via raising iron, using an observational and Mendelian randomization study.

Methods
To clarify the observational study please address the following points
1.      Please ensure that the analysis is adjusted comprehensively for confounders. Confounders are typically common causes of exposure and outcome, as explained here. Socio-economic position is often an influential confounder that likely affects many estimates not just those concerning education

We thank the reviewer for this suggestion. The observational analyses are now corrected for Townsend Deprivation Index, historical job and total household income; the inclusion of which did not substantially alter our overall conclusions.

2.      Please ensure the analysis is not adjusted for consequences of the exposure unless they are independent of the outcome. Adjusting for non-confounders can bias estimates as explained here [https://pubmed.ncbi.nlm.nih.gov/16931543/](https://pubmed.ncbi.nlm.nih.gov/16931543/).

Covariates have been selected on this basis. We now include a sensitivity analyses excluding blood pressure and cholesterol (**STable 3**), which although we would argue are potential confounds, have been previously associated with heavy alcohol intake. This did not alter the results. We cite this paper in the methods.

3.      When assessing interactions, please ensure interactions with confounders are included in the model, as explained here [https://pubmed.ncbi.nlm.nih.gov/22422832/](https://pubmed.ncbi.nlm.nih.gov/22422832/)

As our models have >500 image-related confounds, we have tested for interactions with a parsimonious number of sociodemographic factors (age, sex, smoking and

Townsend Deprivation Index). Only age interactions (for bilateral putamen and caudate) were significant after multiple testing correction. We now explain this in the text, and provide the age and sex interactions for all brain regions in **STable 3**, instead of showing the putamen susceptibility associations by sex. We comment on this in the results:

*“There were significant interactions with age in bilateral putamen and caudate* ***(STable 3****), but not sex, smoking or Townsend Deprivation Index.”*

4.      Please explain how the issue of the exposure starting before recruitment may affect the estimates as explained here [https://pubmed.ncbi.nlm.nih.gov/27237061/](https://pubmed.ncbi.nlm.nih.gov/27237061/)

We have added left truncation as a potential limitation to the discussion:

“*Alcohol exposure prior to study baseline (left truncation) may bias observational estimates [78].”*

5.      Please provide tables showing exposures by potential confounders so that the reader can assess the likely level of confounding

We have added **STable 2** showing potential confounders according to different alcohol intakes, and include the following in the results:

“*Characteristics according to alcohol consumption are shown in* ***STable 2****. Never drinkers comprised a higher proportion of females, lower blood pressure, and higher prevalence of diabetes compared to higher intake alcohol consumers.”*

6.      Please also provide a table showing how the sample with brain and liver imaging relates to UK Biobank on key characteristics, so that the reader can assess any issues arising from selection

We have added a column to **Table 1** to show how the two imaging samples compare to the wider UKB sample.

As regards the Mendelian randomization (MR) study, please clarify the following points in the manuscript
1.      Were the SNPs used as genetic instruments independent?

We now clarify the SNPs were independent:

*“Genetically predicted alcohol consumption was instrumented using 91/92 [depending on SNP or suitable proxy availability in outcome data]* ***independent*** *SNPs…..*

*Alcohol use disorder (AUD) was instrumented using 24 conditionally* ***independent*** *genome-wide significant genetic variants in the largest published GWAS…”*

2.      Were the SNPs used as genetic instruments for iron independent of hepcidin and hereditary hemochromatosis?

There is overlap between SNPs associated with serum iron, hereditary haemochromatosis, liver and brain T2* (particularly HFE). As far as we know, there have not been any specific GWAS of hepcidin that have found variants passing genome-wide significance.

3.      Did the inverse variance weighted estimates use multiplicative effects?

Yes – we now clarify this in the text:

*“Inverse variance weighted (IVW) analysis (multiplicative random effects) regresses…”*

4.      Please include power calculations

We now give the power for MR analyses in our discussion of limitations:

*“Therefore our analysis within the imaging sample, despite its large size (n=29,579) had 65.4% power to detect a causal effect of 0.25 S.D. (based on R^2^=0.003 and α=0.05). The power for the larger serum iron measures was greater (>80% to detect a causal effect of 0.13 S.D.).”*

We include details about how the power calculations were performed in the methods:

*“Power calculations for MR analyses were based on an online calculator developed by one of the authors (https://sb452.shinyapps.io/power/).”*

5.      Would it be possible to use multivariable MR to show that the effect of alcohol on cognition is mediated by iron?

We agree this is an interesting avenue, and plan to include this in a future cognition-focused manuscript. We currently only have ~65% power to detect a “direct” association, so while interesting, this will not be feasible until further imaging data is released.

Discussion

To contextualize the results please explain whether any other drivers of iron levels, such as supplements or foods rich in iron, would be expected to, or are known to, have detrimental effects on cognition. Similarly, iron levels vary by sex and population, are corresponding differences in cognition seen?

We have added these suggestions to the discussion as follows:

*“Although most studies of dietary iron and cognition have been in children or anaemic individuals, there is some evidence that high dietary iron associates with poorer cognition [70].”*

*“Whilst sex differences in cognition have been described [71], it is difficult to disentangle differing iron levels from hormonal factors in the aetiology.”*

In the interpretation, please consider whether the changes with age could be a cohort effect. The UK Biobank participants encompass a wide age range recruited over a short time span, so age also represents cohort.

We have added this as a discussion point:

*“We are mindful however that within UKB, changes with age could represent a cohort effect.”*

Please give more thorough consideration of the limitations of the methods used.

We have expanded upon the limitations substantially in response to the feedback of the handling editor.

**Reviewer #2:** In this new manuscript, Topiwala et al report an association between alcohol consumption and MRI-determined brain iron. This study has public health importance, since the association was observed in even moderate drinkers. The importance of brain iron to neurological diseases is increasingly appreciated, and a proposed model of iron elevation mediating alcohol-related cognitive deficits is plausible. The study examined a large and well characterized dataset (n>22k) of brain MR images, and combined this with additional genetic data, clinical characteristics (including several risk factors), cognitive outcomes, and systemic values of iron from plasma and liver MRI. The authors note a major, but almost unavoidable limitation of this dataset is that alcohol use is self-reported, but otherwise this represents an ideal dataset to perform this study on. This limitation is somewhat mitigated by the use of genetic predictors of alcohol use disorder, where the authors
report that these too are associated with brain iron in certain regions. Overall, I found this to be a well conducted, comprehensive, and important study. I have the following comments:

*       Age and sex are likely to influence brain iron. I note that the authors controlled for these variables, but it is perhaps likely that the association between alcohol use and brain iron may be non-linear with age, or may be more apparent in either males or females. Is an interaction term of age*alcohol or sex*alcohol significant? Or are there different results when comparing associations in strata of sex or age (young/old). It may be of public health importance to know whether, for example, the association only occurs after a certain age, or only in males.

Thank you for this suggestion. We now report interactions between alcohol and age/sex, and non-linear terms for alcohol consumption in the results. As we now report, there is a significant interaction between alcohol and age in terms of putamen and caudate susceptibility.

*       The figures were distorted when printed to the PDF. There appear to be random boxes covering parts of the figures, and other significant graphical issues.

Apologies for this. We have now submitted separate image files.

*       In Figure 2, the colours of the dots are not explained. The inset is hard to read and should be included as a separate table.

The colour scheme is now explained. The insert has been enlarged and presented separately (**Figure 3**).

*       The authors make the case for alcohol use causing brain iron elevation. I think this is likely, but would the authors consider also the reverse possibility - that people with high brain iron are more likely to drink alcohol. A possible mechanism for this is that tyrosine hydroxylase (dopamine-producing enzyme) is dependent on iron. 

Thank you for this interesting hypothesis. We include discussion of it in the paper:

*“The alternative possibility is that individuals with higher brain iron drink more alcohol. One potential mechanism for this is that tyrosine hydroxylase, an enzyme in the dopamine synthesis pathway, is iron-dependent [59]. Dopamine has been linked to alcohol cravings in dependence [60]. For this reason we used MR to support/refute the observational analyses.”*

Scott Ayton


**Reviewer #3:** The authors use observational and Mendelian randomization (MR) analyses to investigate whether moderate alcohol consumption leads to higher levels of iron concentration in various brain regions, whether this effect of alcohol on brain iron is mediated by systemic iron levels, and whether higher concentration of brain iron causes a sharper decline in age-related cognitive function.

The manuscript uses suitable large-scale datasets, makes use of appropriate methodology, links the obtained results with existing literature and discusses the biological plausibility and clinical relevance of novel findings. I enjoyed reading the manuscript and believe it is already deserving of publication. I have only a few minor comments:

Thank you for the positive comments.

1) The effects of alcohol consumption on health-related traits are sometimes non-linear. At the same time, the authors state that part of their motivation for conducting this study is to consider the impact of moderate alcohol consumption on brain iron levels, as opposed to heavy consumption which has already been investigated in the literature. With that in mind, did the authors observe any evidence of non-linear behaviour in the effects of alcohol consumption on brain iron levels and cognitive function?

We agree and categorized alcohol intake in the observational analyses into quintiles as one method to investigate non-linear patterns. We now additionally specifically state that we tested quadratic terms for alcohol:

*“Interactions between alcohol and age and sex were explored, as well as quadratic terms for alcohol to examine for non-linear effects of alcohol.”*

And comment that these were not significant for brain susceptibility:

*“Quadratic terms for alcohol were not significant.”*

But were for cognitive function:

*“In a post-hoc analysis, squared alcohol consumption was associated with slower TMT performance (alcohol β =-4.09x10^-3^, 95% CI: -0.02 to 0.01, p=0.53; alcohol^2^ β= 0.01, 95% CI: 3.84x10^-3^ to 0.018, p=0.003)..”*

Given the weak associations (probably as a result of low power as discussed in limitations) in MR, we did not pursue non-linear MR analyses.

2) The authors mention that the genetic variants used in their study explained less than 1% of variation in alcohol consumption. Does that mean their MR analysis could be susceptible to weak instrument bias? Since the paper uses a two-sample design, any such bias would act towards the null, making it harder to identify significant associations between drinking and iron levels. Perhaps it would be a good idea to report F-statistics as an indication of instrument strength in the authors' MR analyses.

Yes we agree weak instrument bias is a potential limitation. Although we do not have individual data for the GSCAN cohorts, we now report F-statistics from European UKB subjects and include discussion of this in the limitations:

*“Furthermore, weak instrument bias, in the direction of the null may be contributing [50]. Using UK Biobank for our calculations, about one third of SNPs we used to instrument alcohol consumption had F statistics <10 (****STable 10****).”*

3) There must be an issue with the confidence intervals plotted in Figure 6, as the "+1 SD" and "-1 SD" lines seem to be reversed for smaller ages.

Thank you for highlighting that the legend for Figure 6 (now **Figure 7**) was not clear. We are plotting an interaction, the susceptibility-dependent effect of age on TMT.  The multiple lines are not confidence intervals, but the age-TMT fit for 3 different values of putamen susceptibility (mean-SD, mean, and mean+SD); with this interaction the reviewer has correctly observed that the impact of putamen susceptibility is reversed for young and old. The figure legend has been revised for clarity.

4) Directly below Figure 2, a few lines of text have been duplicated - please delete.

These have been removed.

**Reviewer #4:** This is a comprehensive, well-conducted and clearly written examination of the relationship between brain iron and alcohol use. The study includes robust methods and appropriate sensitivity analyses, leading to convincing evidence that low levels of alcohol use are associated with iron accumulation in the brain. Given the prevalence of low-level alcohol use, the findings have considerable public health implications, particularly in terms of improving cognitive health. There are some details we feel should be addressed:

Thank you.

1)      The models predicting alcohol use did not include sociodemographic covariates which were included in the models with cognitive outcomes. These covariates seem relevant to the alcohol analyses as well and we wondered why they weren't included? It was also unclear why the models investigating the relationship with serum iron (figures S13 and 14) then included the sociodemographic factors?

All analyses are now reported from models including sociodemographic covariates (Townsend Deprivation Index, household income and historical job code).

2)      What was the motivation for including the analysis of genetically predicted AUD? Much of the introduction focuses on moderate levels of alcohol use and the importance of understanding the impact of this from a public health perspective so the inclusion of AUD is surprising. In addition, the statistical analysis section focusing on MR only mentions alcohol consumption not AUD.

Whilst our main focus was moderate alcohol consumption, we included AUD for the MR analysis as there has not been, to our knowledge, previous MR investigation of AUD and serum iron or brain iron. We have now clarified that AUD was also examined in the statistical analysis section.

3)      When describing figures S2-6 the authors state: "Levels of drinking necessary to observe higher susceptibility values differed according to region". It may be worth noting in the text that drinking 7+ units was associated with higher susceptibility for all regions.

This sentence has been amended as suggested to:

*“Drinking greater than 7 units weekly was associated with higher susceptibility for all brain regions, except the thalamus.”*

4)      For sensitivity analyses which stratified the sample by sex and included diet-related covariates, only results for left putamen are shown. This is fine, but please confirm in text that only this is shown for illustrative purposes and that the analyses were conducted for all IDPs and that the pattern of relationships was similar across IDPs (if this is the case). Please also provide n for the sample including diet-related factors.

As requested by the editors, we now include, for all brain regions, analyses with age and sex interactions (**STable 3**), as well as estimates adjusted for diet-related covariates in the supplementary materials (**STable 7**). We also give numbers for all analyses in a flowchart (**SFigure 1**), and baseline characteristics for the sample with complete data on diet and iron supplementation (**STable 6**).

5)      In terms of categorisation of alcohol consumption, the categories overlap slightly (i.e., 7-12, 12-18 etc). Please confirm this is a labelling error and correct if this is the case. The analyses for QSM and T2* seem to have alcohol categorised slightly differently - is there a reason for this?

We have amended the labeling to clarify there was no overlap between alcohol categories and consistent categorization for QSM and T2* analyses.

6)      What is the sample size for the serum iron analyses?

We now give sample sizes as a supplementary table (**STable 1**) detailing the cohorts used in MR analyses.

7)      Can the authors please provide more detail on their causal mediation analysis and how what is presented is different to standard mediation analysis.

We have expanded our description of causal mediation analyses in the methods section as explained in response to an earlier comment. We also specifically comment on the advantages of CMA over traditional mediation analyses:

*“CMA, unlike traditional mediation methods, defines, identifies, and estimates causal mediation effects without reference to a specific statistical model…….CMA was run on participants with complete data. Two separate multiple linear regression models were run. First, with putamen susceptibility as the dependent variable, with alcohol, age, sex, smoking, blood pressure, iron genetic risk score, BMI, cholesterol and liver iron as covariates. In the second model, the mediator, liver T2*, was the dependent variable, with all covariates as in the first model. CMA was then run using nonparametric bootstrapping to generate confidence intervals (1000 simulations). Assumptions in CMA include no unmeasured confounding between the exposure and outcome, between the exposure and mediator, or between the mediator and the outcome.”*

8)      Given the sample size, one-sample non-linear MR would not have been possible. But given that many studies do find a non-linear relationship between alcohol use and health outcomes (specifically a protective effect of low consumption) consideration of this as a limitation may be included.

We agree non-linear relationships would be interesting (and now include non-linear results for our observational analysis), and we have applied these within the larger UKB sample. However, for this analyses, limited to the imaging sub-sample, we agree there is insufficient power for a non-linear MR. We now include this in the limitation section:

*“Therefore our analysis within the imaging sample, despite its large size, was not powered to detect small associations with alcohol. For this reason, although non-linear relationships between alcohol and health outcomes are of interest, we limited MR analyses to linear models.”*

Overall, we would like to commend the authors on an impressive piece of work.

Thank you.

Dr Louise Mewton & Ms Rachel Visontay
Centre for Healthy Brain Ageing, University of New South Wales, Sydney, Australia
